# Associations between bolus infusion of hydrocortisone, glycemic variability and insulin infusion rate variability in critically Ill patients under moderate glycemic control

**DOI:** 10.1186/s13613-015-0077-5

**Published:** 2015-11-02

**Authors:** Roosmarijn T. M. van Hooijdonk, Jan M. Binnekade, Lieuwe D. J. Bos, Janneke Horn, Nicole P. Juffermans, Ameen Abu-Hanna, Marcus J. Schultz

**Affiliations:** Department of Intensive Care Medicine, Academic Medical Center, University of Amsterdam, Meibergdreef 9, 1105 AZ Amsterdam, The Netherlands; Laboratory of Experimental Intensive Care and Anesthesiology (L·E·I·C·A), Academic Medical Center, University of Amsterdam, Amsterdam, The Netherlands; Department of Medical Informatics, Academic Medical Center, University of Amsterdam, Amsterdam, The Netherlands

**Keywords:** Hydrocortisone, Bolus infusion, Blood glucose control, Glycemic control, Blood glucose variability, Insulin, Insulin infusion rate variability

## Abstract

**Background:**

We retrospectively studied associations between bolus infusion of hydrocortisone and variability of the blood glucose level and changes in insulin rates in intensive care unit (ICU) patients.

**Methods:**

‘Glycemic variability’ and ‘insulin infusion rate variability’ were calculated from and expressed as the standard deviation (SD) of all blood glucose levels and insulin infusion rates during stay in the ICU, respectively. Glycemic and insulin infusion rate variability in patients who received bolus infusion of hydrocortisone were compared to those in patients who never received bolus infusion of hydrocortisone. Multivariate analysis was performed to correct for potential covariates including disease severity.

**Results:**

We included 6409 patients over 6 years; of them 962 received bolus infusion of hydrocortisone. Compared to patients who never received bolus infusion of hydrocortisone, patients who received hydrocortisone had their blood glucose level measured more frequently, had higher glycemic variability; were more frequently treated with intravenous insulin and had higher insulin infusion rate variability. The association between hydrocortisone treatment and glycemic variability was independent of disease severity, but the effect of hydrocortisone treatment on blood glucose variability was less strong in the more severely ill patients. The association between hydrocortisone and insulin infusion rate variability was also independent of disease severity, and independent of glycemic variability.

**Conclusions:**

Bolus infusion of hydrocortisone is independently associated with higher glycemic variability and higher insulin infusion rate variability in ICU patients. Studies are needed to see if continuous infusion of hydrocortisone prevents higher glycemic variability and higher insulin infusion rate variability.

**Electronic supplementary material:**

The online version of this article (doi:10.1186/s13613-015-0077-5) contains supplementary material, which is available to authorized users.

## Background

Low-dose hydrocortisone treatment is an accepted therapy for patients with refractory shock [1]. Bolus infusion of hydrocortisone, however, could induce short episodes of hyperglycemia frequently requiring temporary adjustments of the insulin infusion rate in critically ill patients [2, 3]. While experienced intensive care unit (ICU) nurses usually are capable of preventing large swings in the blood glucose level [4], they could be less skilled in avoiding hydrocortisone-induced dysglycemia. Furthermore, preventing hydrocortisone-induced dysglycemia could largely increase nurse labor and costs associated with blood glucose monitoring, as temporary adjustments of insulin infusion rates could also increase the frequency of blood glucose measurements [3, 5]. Notably, glycemic variability is associated with increased mortality in critically ill patients [6].

It is unknown to what extent bolus infusion of hydrocortisone increases glycemic variability and adjustments in insulin infusion rates in patients under moderate glycemic control. Therefore, in a cohort of patients receiving blood glucose control aiming at blood glucose levels between 90 and 144 mg/dL, we tested the two following hypotheses: (a) bolus infusion of hydrocortisone is associated with glycemic variability, and (b) bolus infusion of hydrocortisone is associated with insulin infusion rate variability.

## Methods

### Study design

This was a retrospective cohort study performed in a 32-bed mixed medical-surgical ICU in a university hospital in the Netherlands (the Academic Medical Center, Amsterdam, The Netherlands). The Institutional Review Board approved the study protocol and waived the need for individual patient consent or ethical approval to collect and analyze data from registries that exclude patient-identifying information.

### Study setting

The ICU was a closed-format unit with patients under the direct care of a team of board-certified ICU nurses and physicians. The nurse to patient ratio was 1:2. All beds were equipped with the MetaVision^®^ patient data management system (iMDsoft, Tel Aviv, Israel) in which all blood glucose levels and insulin infusion rates were stored automatically.

### Blood glucose control

The local guideline for moderate blood glucose control did not change during the period of data collection, and has been described before [7–9]. In short, experienced ICU nurses carefully titrated insulin aiming at a blood glucose level between 90 and 144 mg/dL. For this, continuous insulin infusion was started when the blood glucose level exceeded 144 mg/dL, and adjustments were made following a flow chart (see Additional file 1). Insulin infusion was stopped, and a bolus of 50 mL dextrose 20 % was given when the blood glucose level dropped <63 mg/dL. The flow chart provided recommendations regarding timing of follow-up blood glucose measurement, which could vary from 20 min to 4 h. Blood glucose levels were exclusively measured in blood samples obtained via an arterial catheter using a RapidLab 1265 blood gas analyzer (Siemens Healthcare Diagnostics, The Hague, The Netherlands) located in the ICU.

### Indications for and protocol of hydrocortisone therapy

Indications for bolus infusions of hydrocortisone did not change during the period of data collection. ICU physicians prescribed bolus infusion of hydrocortisone in patients with refractory shock, defined as shock that was non-responsive to fluid resuscitation and poorly responsive to vasopressor therapy. Bolus infusion of hydrocortisone started at 100 mg every 8 h for 1 week, after which it was slowly tapered, according to international guidelines [10]. Bolus infusion of hydrocortisone was also initiated in patients with proven or suspected adrenal insufficiency, e.g., patients who received longstanding treatment with glucocorticosteroids before ICU admission.

### Study population

The study cohort consisted of patients admitted to the ICU during a six-year period, lasting from January 2007 to December 2012. Patients <18 years old, readmitted patients and patients who were discharged alive within 24 h were excluded from the analysis. Furthermore, we excluded patients who had their blood glucose level measured fewer than three times during the entire stay in the ICU. Finally, patients who received treatment with glucocorticosteroids other than hydrocortisone, and patients who received continuous infusion instead of bolus infusion of hydrocortisone were excluded.

### Data collected

Blood glucose levels, insulin infusion rates and boluses of hydrocortisone were extracted from the patient data management system. Demographic data were extracted from the National Intensive Care Evaluation (NICE) database, a validated database with high quality of data maintained by the NICE Foundation [11], including gender, age, length, weight, admission diagnosis, admission type, the Acute Physiology and Chronic Health Evaluation (APACHE) II score [12], ICU length of stay, hospital length of stay, ICU and hospital mortality.

### Primary and secondary endpoints

The primary endpoint was glycemic variability. The secondary endpoints were the insulin infusion rate variability and the number of blood glucose measurements during stay in ICU. Glycemic variability was calculated from and expressed as the SD of all blood glucose levels per patient over the entire stay in ICU, as described before [6, 13] and as recommended in the consensus guidelines [14]. We chose to use the SD, as this is the most frequently used metric for glycemic variability. Insulin infusion rate variability was calculated from and expressed as the SD of insulin infusion rates per patient over the entire stay in ICU.

### Power analysis

We did not perform a power calculation, as this was a retrospective observational exploratory analysis. We assumed to have sufficient numbers of patients when including the whole cohort over a period of 6 years.

### Analysis plan

Demographic, blood glucose and insulin metrics were summarized and compared between patients who did and did not receive a bolus infusion of hydrocortisone during ICU admission. Data were compared using the Student’s *t* test, the Mann–Whitney *U* test or the Chi-Square test. Statistical significance was considered to be at a *P* value <0.05. When appropriate, statistical uncertainty was expressed by the 95 % confidence levels.

We used univariate and multivariate linear regression analysis to test whether bolus infusion of hydrocortisone was independently associated with increased glycemic variability and insulin infusion rate variability. For this, glycemic variability was logarithmically transformed (using the natural logarithm) to obtain a more normal distribution. We categorized insulin infusion rate variability to ‘no insulin infusion rate variability’ (which included patients who never received insulin during the entire stay in ICU, and patients with fewer than three changes in the insulin infusion rate), and the three tertiles of the SD of the insulin infusion rate.

Thus, two separate multivariate models were developed, one model for glycemic variability and one model for insulin infusion rate variability. We first investigated if disease severity, expressed as the APACHE II score, was a significant interaction term with hydrocortisone. We stratified for the APACHE II scores into three groups [15, 16], when the *P* value of the interaction term was significant and the model fit improved (based on Akaike Information Criterion). The models included variables that were established as confounders [17]. A confounder was defined as a variable that is not on the casual path between hydrocortisone infusion and glycemic or insulin variability. Thus, for instance, we did not include vasopressor use as we considered it likely that vasopressor use is on the casual path. The following variables were tested for confounding: gender, BMI and type of admission (non-surgical, elective surgery, emergency surgery). All these variables met the theoretical criteria for confounding because they are (surrogate) causes or risk factors of outcome; and associated with the exposure (but not affected by it) [17].

Correlation between covariates was assessed to investigate collinearity. Pearson correlation coefficients were all under 0.5 showing no significant collinearity. The effect of covariates on the glucose variability was reported as the percentage of change in the glucose variability with the confidence interval. The effects of covariates on the insulin infusion rate variability were reported as odds ratios with the 95 % confidence interval. This odds ratios represent the odds of having a high insulin infusion rate variability (the highest tertile of the SD) compared to having no variability, or a small or moderate insulin infusion rate variability (the lowest and middle tertile of the SD), the odds of having moderate and high insulin infusion rate variability versus no or small insulin infusion rate variability, and the odds of having small, moderate and high insulin infusion variability rate versus having no variability. Finally, an ordinal regression was made to test if bolus infusion of hydrocortisone, independently of glycemic variability, was associated with insulin infusion rate variability.

In a post hoc analysis, we used the glycemic lability index (GLI) for glycemic variability, as this metric for glycemic variability takes into account the time interval between measurements [18]. The median GLI per day per patient was used in the analysis.

Diabetic status can be a potential confounder, but diabetic status was not captured, or captured incompletely, in the first 2 years of the cohort. We performed a second post hoc analysis, including only patients in whom diabetic status was reliably captured.

Analyses were performed using R (version: 3.1.1; R Foundation for Statistical Computing, Vienna, Austria).

## Results

### Patients

**Fig. 1:**
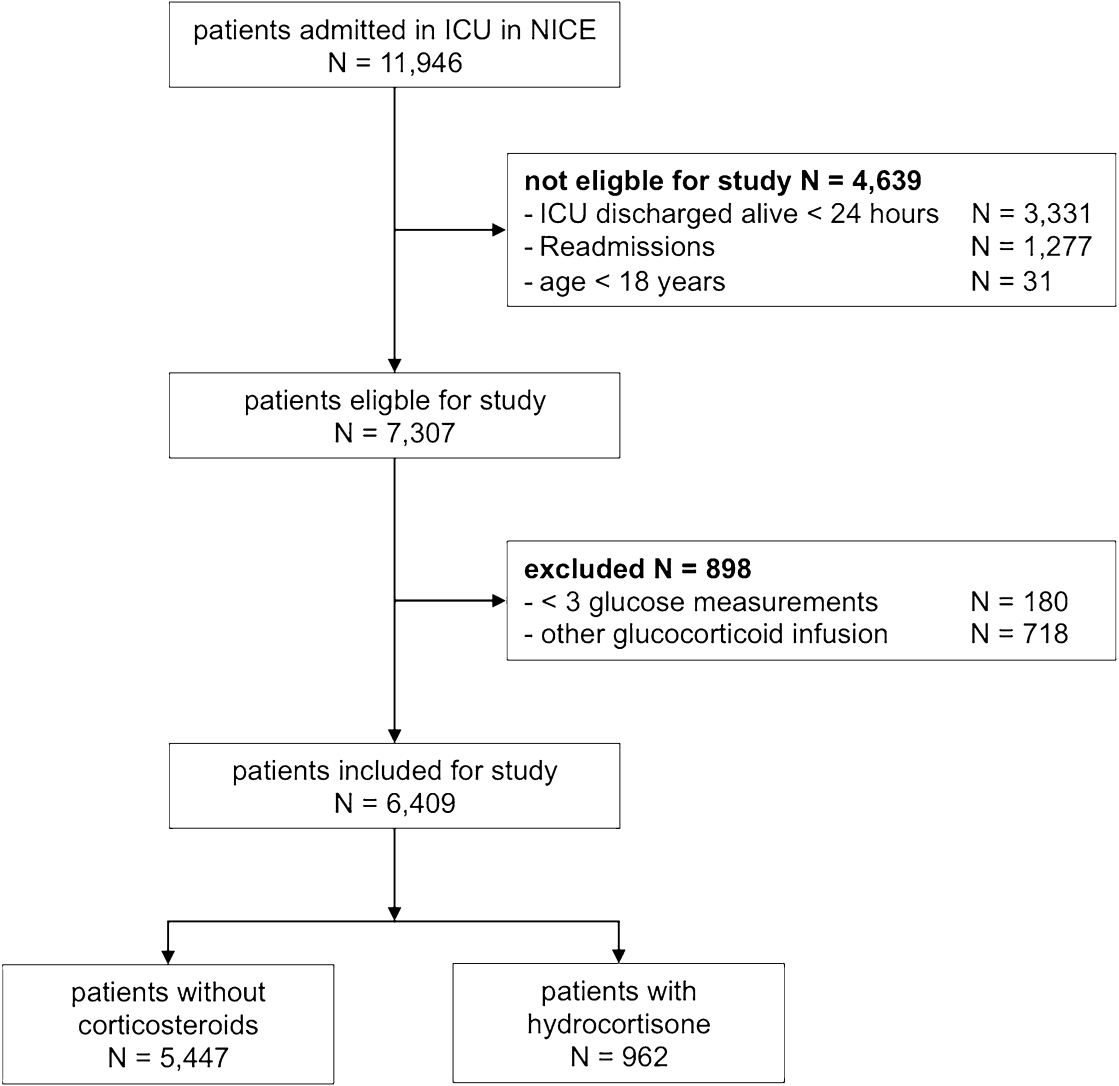
CONSORT diagram of the study

Of 11,946 patients 4638 met the exclusion criteria for the present analysis (Fig. 1). One hundred eighty patients had fewer than three blood glucose measurements available, necessary to calculate glycemic variability, and 718 patients received treatment with other glucocorticosteroids than hydrocortisone. Continuous infusion of hydrocortisone was never used. Table 1 shows demographic data of the remaining 6409 patients. Patients treated with hydrocortisone were older, more severely ill according to APACHE II scores, more often non-surgical patients and died more frequently.

**Table 1:** Patient characteristics

Characteristics	Patients who did not receive bolus infusion of hydrocortisone (N = 5447)	Patients who received bolus infusion of hydrocortisone (N = 962)	P value	Total cohort (N = 6409)
Age (years, median [IQR])	63 [51–73]	65 [55–74]	<0.001	63 [52–73]
Male gender [no (%)]	3431 (63.0)	571 (59.4)	0.035	4002 (62.4)
BMI (kg/m^2^, median [IQR])	25 [23–28]	25 [23–29]	0.968	25 [23–28]
Admission diagnosis [no (%)]			<0.001	
Non-surgical	2159 (39.6)	542 (56.3)		2701 (42.1)
Emergency surgery	993 (18.2)	200 (20.8)		1193 (18.6)
Elective surgery	2295 (42.1)	220 (22.9)		2515 (39.2)
History of diabetes [no (%)]				
Diabetes	550 (14.7)	102 (16.5)	0.281	652 (15.0)
Non diabetes	3188 (85.3)	517 (83.5)		3705 (85.0)
Missing values	1709	343		2052
APACHE II scores (median [IQR])	18 [14–24}	24 [19–30]	<0.001	19 [14–25]
SAPS II score	40 [31–52]	55 [44–67]	<0.001	42 [32–54]
ICU LOS (days, median [IQR])	2 [1–4]	6 [3–12]	<0.001	3 [2–5]
Hospital LOS (days, median [IQR])	10 [6–20]	16 [8–36]	<0.001	11 [6–22]
ICU mortality [no (%)]	658 (12.1)	306 (31.8)	<0.001	961 (15.0)
Hospital mortality [no (%)]	944 (17.3)	391 (40.6)	<0.001	1335 (20.8)

*APACHE* acute physiology and chronic health evaluation, *BMI* body mass index, *ICU* intensive care unit, *IQR* interquartile range, *LOS* length of stay

### Blood glucose metrics and insulin infusion metrics

Hypoglycemia and hyperglycemic measurements were more frequently found in patients treated with bolus infusions of hydrocortisone (Table 2). Glycemic variability was higher in patients who received hydrocortisone. The total number of blood glucose measurements during stay in ICU, and the median number of blood glucose measurements per day was higher in patients who received hydrocortisone.

**Table 2:** Blood glucose metrics

	Patients who did not receive bolus infusion of hydrocortisone (N = 5447)	Patients who received bolus infusion of hydrocortisone (N = 962)	P value	Total cohort (N = 6409)
Number of measurements (median [IQR])	20 [13–35]	53 [30–97]	<0.001	22 [13–42]
Median number of measurements per day (median [IQR])	6 [5–8]	7 [6–9]	<0.001	6 [5–8]
Median number of measurements per day with hydrocortisone (median [IQR])		8 [7–10]		
Median number of measurements per day without hydrocortisone (median [IQR])		6 [4–7]		
Mean blood glucose level (mg/dL, median [IQR])	136 [125–148]	136 [127–148]	0.043	136 [126–148]
Median blood glucose level (mg/dL, median [IQR])	132 [122–144]	132 [123–141]	0.588	132 [123–144]
Standard deviation of blood glucose level (mg/dL, median [IQR])	28 [20–39]	35 [26–47]	<0.001	29 [21–40]
Glycemic Lability Index (mg/dL^2^/hour/day median [IQR])	942 [371–2236]	1322 [594–2883]	<0.001	994 [400–2369]
Maximum blood glucose level (mg/dL, median [IQR])	193 [166–234]	231 [193–285]	<0.001	198 [169–241]
Minimum blood glucose level (mg/dL, median [IQR])	90 [77–101]	70 [56–85]	<0.001	88 [74–99]
Mild hypoglycemia, <70 mg/dL (%)	826 (15.2)	451 (46.9)	<0.001	1277 (19.9)
Severe hypoglycemia, <40 mg/dL (%)	115 (2.1)	80 (8.3)	<0.001	195 (3.0)
Hyperglycemia, >180 mg/dL (%)	3438 (63.1)	824 (85.7)	<0.001	4262 (66.5)

*IQR* interquartile range, *IU* international units

Patients treated with bolus infusions of hydrocortisone were treated with insulin more frequently (Table 3). They received more boluses of insulin, and had more frequent adjustments in the insulin infusion rate. Consequently, they had higher insulin infusion rate variability.

**Table 3:** Insulin metrics

	Patients who did not receive bolus infusion of hydrocortisone (N = 5447)	Patients who received infusion of hydrocortisone (N = 962)	P value	Total cohort (N = 6409)
Patients receiving insulin infusion [no (%)]	3693 (67.8)	875 (91.0)	<0.001	4568 (71.3)
Patients receiving boluses of insulin infusion [no (%)]	490 (9.0)	248 (25.8)	<0.001	738 (11.5)
Number of insulin rate adjustments (median [IQR])	3 [0–8]	17 [6–35]	<0.001	4 [0–12]
Median number of insulin rates per day (median [IQR])	1 [0–2]	2 [1–3]	<0.001	1 [0–2]
Median number of insulin rates per day with hydrocortisone (median [IQR])		3 [1–4]		
Median number of insulin rates per day without hydrocortisone (median [IQR])		1 [0–2]		
Patients in insulin infusion rate variability category:				
No insulin infusion rate variability	2494 (45.8)	136 (14.1)		2630 (41.0)
Small insulin infusion rate variability	887 (16.3)	111 (11.5)		998 (15.6)
Moderate insulin infusion rate variability	1104 (20.3)	260 (27.0)		1364 (21.3)
High insulin infusion rate variability	962 (17.7)	455 (47.3)		1417 (22.1)
Standard deviation of insulin rate (IU/h, median [IQR])	0.6 [0–1.3]	1.6 [0.8–6.2]	<0.001	0.7 [0–1.6]

*IQR* interquartile range, *IU* international units

### Univariate and multivariate analyses

Tables 4 and 5 detail the results for the univariate and multivariate analyses. For all the three models, APACHE II score was a significant interaction term and therefore the analysis was stratified per APACHE II score category. In all three APACHE II score categories, bolus infusion of hydrocortisone was independently associated with higher glycemic variability expressed as the SD, though the effect was less strong in more severely ill patients.

**Table 4:** Results of univariate and multivariate analysis with glycemic variability

Variable	Univariate		Multivariate	
	Percentage of change in glycemic variability [95 % CI]	P value	Percentage of change in glycemic variability [95 % CI]	P value
APACHE II score <15				
Bolus infusion of hydrocortisone	20 [5 – 36]	0.008	23 [8 – 40]^*a*^	0.0018
APACHE II score 15–24				
Bolus infusion of hydrocortisone	22 [16 – 29]	<0.001	23 [16 – 30]^*b*^	< 0.001
APACHE II score >24				
Bolus infusion of hydrocortisone	15 [8–21]	<0.001	14 [8–20]^*c*^	< 0.001

*APACHE* acute physiology and chronic health evaluation, *BMI* body mass index, *CI* confidence interval

^*a*^Multivariate model includes the significant confounders Admission Type, and BMI

^*b*^Multivariate model includes the significant confounders Admission Type, gender and BMI

^*c*^Multivariate model includes the significant confounders Admission Type, and gender

**Table 5:** Results of univariate and multivariate analysis with insulin infusion rate variability

Variable	Univariate	Multivariate	Multivariate including glycemic variability
	Odds ratio [95 % CI]	Odds ratio [95 % CI]	Odds ratio [95 % CI]
APACHE II score <15			
Bolus infusion of hydrocortisone	3.8 [2.4–6.0]	4.2 [2.6–6.7]^*a*^	4.1 [2.5–6.7]
APACHE II score 15–24			
Bolus infusion of hydrocortisone	4.0 [3.3–4.9]	4.0 [3.3–4.9]	3.6 [2.9–4.4]
APACHE II score >24			
Bolus infusion of hydrocortisone	3.1 [2.6–3.7]	3.1 [2.6–3.7]	3.1 [2.5–3.7]

*APACHE* acute physiology and chronic health evaluation, *BMI* body mass index, *CI* confidence interval

^*a*^Multivariate model includes the significant confounder BMI

Patients who received bolus infusion of hydrocortisone had higher odds of having higher insulin infusion rate variability, independently of confounders. The association between bolus infusion of hydrocortisone and insulin infusion rate variability remained statistically significant when glycemic variability was included as confounder.

### Post hoc analyses

In the first post hoc analysis, bolus infusion of hydrocortisone was independently associated with higher GLI in the group of patients with APACHE II score between 15 and 24 (Additional file 1: Table S1). Entering GLI as confounder between bolus infusion of hydrocortisone and insulin infusion rate variability did not affect the association (Additional file 1: Table S2).

In the second post hoc analysis, including only patients in whom diabetic status was reliably captured, the association between bolus infusion of hydrocortisone and glycemic and insulin variability remained statistically significant when diabetic status was included as confounder (Additional file 1: Tables S3, S4).

## Discussion

The results of this retrospective analysis of blood glucose levels and insulin infusion rates in ICU patients under moderate glycemic control show that bolus infusion of hydrocortisone is associated with higher glycemic variability and insulin rate variability, more frequent BG measurements and a greater need for insulin boluses. These associations were independent of several factors, including severity of disease and also diabetic status. One salient finding in the present analysis was that the associations between bolus infusions of hydrocortisone and glycemic variability and insulin infusion rate variability were less strong in patients who were more severely ill. It could be that the impact of severity of disease on variability of the blood glucose level and the insulin infusion rate is much stronger than the impact of bolus infusions of hydrocortisone. This could partly blur the associations, which we were interested in. Furthermore, when GLI was used to express glycemic variability, the independent association between bolus infusion and glycemic variability was significant only in patients with an APACHE II score between 15 and 24. The GLI takes into account the time interval and SD does not, which might explain the different results. Still, both measures do not capture all glucose excursions, as we were not continuously measuring the glucose level and thereby missing, by definition, blood glucose excursions between two glucose measurements.

We chose to develop a metric for insulin infusion rate variability, a new metric, based on the standard deviation similar to the one we used for glycemic variability. It should be noticed that insulin infusion rate variability may not reflect changes in insulin resistance or insulin sensitivity, but it could be a measure indicating changes in nursing workload. This was exactly the reason for developing this new metric, as most other metrics in the domain of blood glucose control do not consider workload by nurses. The number of measurements per day was considered before as measure indicating the nursing workload [3]. However, it is not only the blood glucose measurements which is time consuming, also the interpretation of the blood glucose measurement and the change in the insulin infusion pump takes time. In addition, trained nurses are necessary to perform the blood glucose control or when available decision support could be used.

The results are, at least in part, in line with those from several previous studies. First, in a randomized controlled study in cardiac surgery patients, peroperative treatment with dexamethasone was associated with elevated blood glucose levels for as long as 15 h after surgery, requiring more intensive treatment with insulin [19]. In addition, in a double-blind placebo-controlled randomized study, it was shown that treatment with methylprednisolone was associated with higher blood glucose levels and higher daily insulin doses in patients with COPD exacerbation needing mechanical ventilation [20]. Interestingly, a recent randomized study comparing bolus infusion with continuous infusion of hydrocortisone in septic shock patients showed no differences in the mean blood glucose level, but more hyperglycemic events and more frequent changes in the insulin infusion rate were found in patients receiving bolus infusion of hydrocortisone [3]. Higher glycemic variability was found in patients treated with hydrocortisone, although the difference with patients not treated with hydrocortisone remained statistically insignificant, probably due to the small sample size of fewer than 50 patients. Furthermore, the dosing regimen was different as they used a dose of 50 mg every 6 h opposed to our regimen of 100 mg hydrocortisone every 8 h. It is possible that the higher doses of hydrocortisone in the present study result in greater glucose variability and this might be the reason why we did find an independent association. Our investigation confirms the results of the randomized controlled trial [3] that bolus infusion of hydrocortisone indeed influences glycemic variability and insulin infusion dosing.

It was a practice to infuse a bolus of hydrocortisone every 8 h, and because of its relatively short half-life time of 90 min, we could have expected large swings in blood glucose levels and or insulin infusion rates with this treatment, depending on how the local guideline of glucose control allowed changes in the insulin infusion rate. We were specifically interested in associations between hydrocortisone therapy and glycemic variability, as glycemic variability is considered as one of the domains for quality of blood glucose control and associated with mortality [6, 21]. Previous studies investigating the effect of bolus hydrocortisone on the blood glucose levels in septic shock patients focused on hyperglycemia and not glycemic variability [2, 3, 22]. We are the first to show an independent association between bolus infusion of hydrocortisone and glycemic variability in a large cohort of critically ill patients, though the question remains whether prevention of bolus infusion of hydrocortisone-induced dysglycemia is beneficial. One randomized controlled trial comparing strict blood glucose control with conventional blood glucose control in patients receiving low-dose hydrocortisone for refractory shock did not find clinical benefit from prevention of bolus infusion of hydrocortisone-induced dysglycemia [22]. Notably, this trial did not report metrics of glycemic variability, making it impossible to see whether the intervention in that trial truly influenced glycemic variability. Future studies are necessary to determine whether it is possible to prevent hydrocortisone-induced glycemic variability, and if so whether this improves outcome. There could be a role for continuous glucose monitoring devices that are now slowly entering the market, but this certainly needs confirmation in future studies as well. Notably, a recent retrospective study showed that use of subcutaneous continuous glucose monitoring was not associated with lower glycemic variability in critically ill patients [23].

The results of the present study clearly show that action was required upon hydrocortisone-induced glycemic variability, as the total number of blood glucose level measurements were almost three times as many as in patients not receiving hydrocortisone treatment. Furthermore, adjustments of the insulin infusion rate occurred much more often. While we could not perform a cost analysis, it is clear that costs associated with blood glucose monitoring and adjustments of the insulin infusion rate in patients treated with hydrocortisone exceed those in patients not receiving hydrocortisone. Several studies show that continuous infusion of hydrocortisone is associated with a less steep rise of the blood glucose level than bolus infusion of hydrocortisone [2, 3]. This suggests that continuous infusion of hydrocortisone may need less frequent blood glucose measurements and adjustments of the insulin infusion rate. However, we should bear in mind that continuous infusion of hydrocortisone comes at a price: it may need an additional lumen of a central line or an extra peripheral venous access, but most important it mandates, often expensive, syringe pumps.

Strengths of this study include the large cohort of patients and blood glucose levels without any change in the local guidelines for blood glucose control or indication for bolus infusion of hydrocortisone. Furthermore, the target of the guideline for blood glucose control is a commonly used target in other hospitals, increasing generalizability of the results. Weaknesses of the present study include its retrospective design. Therefore, the data cannot support any causal relationship between steroid treatment and glycemic or insulin variability. In addition, patients receiving boluses of hydrocortisone in the study cohort were almost without exception suffering from septic shock, while most patients not receiving boluses of hydrocortisone were not. Thus, one could argue that differences in blood glucose variability between the two patient groups were simply reflecting differences induced by septic shock. Important in this context is that sepsis may be associated with increased glycemic variability [24]. Indeed, patients with septic shock have increased insulin resistance [25], as also suggested by the other metrics of blood glucose control in our study. However, while insulin resistance is suggested to be dependent on disease severity [25], this was not confirmed here as the effect of hydrocortisone treatment on blood glucose variability was independent of APACHE II scores. The finding that the associations were less strong in more severely ill patients may have been caused by already high blood glucose variability in these patients. Finally, results could have been different when glucocorticosteroids other than hydrocortisone would have been used because half-life times and glucocorticoid effects differ between various glucocorticosteroids agents.

There are several measures to calculate the glycemic variability. We used two measures and found that the association was only significant for both measures in the group of patients with APACHE II score between 15 and 24. It is possible the results will be different when using other measures for glycemic variability [6, 23].

One other major limitation of our analysis is that the frequency of the blood glucose level measurements affects the SD of the blood glucose level [13]. Nevertheless, in previous investigations, glycemic variability remained independently associated with increased mortality [6]. Other limitations of this study are its lack of data regarding nutritional support and corticosteroid usage before ICU admission.

## Conclusions

Bolus infusion of hydrocortisone is independently associated with increased glycemic variability and increased insulin infusion rate variability in ICU patients under glycemic control aiming at blood glucose levels between 90 and 144 mg/dL. Additional studies are needed to investigate if the found associations have an affect on mortality and if continuous infusion of hydrocortisone prevents increased blood glucose variability and insulin infusion rate variability.

## Additional files

10.1186/s13613-015-0077-5 Flow chart for blood glucose control and Tables S1–S4 containing the results for the post hoc analysis.

## Abbreviations

APACHE II score: Acute physiology age and chronic health II score
BMI: Body mass index
ICU: Intensive care unit
IQR: Interquartile range
IU: International units
LOS: Length of stay
NICE: National intensive care evaluation
SAPS–II: Simplified acute physiology score II
SD: Standard deviation
