# Supplementary material for: Associations between bolus infusion of hydrocortisone, glycemic variability and insulin infusion rate variability in critically Ill patients under moderate glycemic control
Source: Ann Intensive Care. 2015 Nov 2;5:34. doi: 10.1186/s13613-015-0077-5 (PMC4630319; doi:10.1186/s13613-015-0077-5)
Supplement: Supplementary file 1 — 10.1186/s13613-015-0077-5 Flow chart for blood glucose control and Tables S1–S4 containing the results for the post hoc analysis. [file 13613_2015_77_MOESM1_ESM.pdf]

Additional file to 'Associations between Bolus Infusion of Hydrocortisone, Glycemic Variability and Insulin Infusion Rate Variability in Critically Ill Patients under Moderate Glycemic Control'

Roosmarijn T.M. van Hooijdonk MD, PhD[1], Jan M. Binnekade PhD[1], Lieuwe D.J. Bos PhD[1,2], Janneke Horn MD, PhD[1], Nicole P. Juffermans MD, PhD[1,2], Ameen Abu–Hanna PhD[3], Marcus J. Schultz PhD[1,2]

Academic Medical Center, University of Amsterdam, Amsterdam, the Netherlands:
[1]Department of Intensive Care Medicine, [2]Laboratory of Experimental Intensive Care and Anesthesiology (L·E·I·C·A), [3]Department of Medical Informatics

Flow chart for blood glucose control

glucose > 360 mg/dL	→	Bolus of 6 units of insulin and start or increase with 4 IU/h	→	Check after 1 hour
glucose 216-360 mg/dL	→	Start or increase with 4 IU/h	→	Check after 1 hour
glucose 144-216 mg/dL	→	Start or increase with 2 IU/h	→	Check after 1 hour
glucose 90-144 mg/dL	→	When decrease of <50% *do not change infusion rate	→	Check after 4 hours
	→	When decrease ≥ 50%*, decrease infusion rate into halve	→	

* from the most recent 2 measurements

Change or stop in nutrition	→	Change or stop insulin infusion always	→	Check after 1 hour[1]
glucose 63-90 mg/dL	→	Decrease ≥ 50 % stop insulin infusion	→	Check after 1 hour[1]
		Decrease < 50 %: decrease infusion rate into halve		Check after 1 hour[1]

[1] when blood glucose level is normoglycemic or hyperglycemic after 1 hour restart insulin infusion with smaller steps (investigate the cause of drop in the blood glucose level)

glucose < 63 mg/dL	→	Stop insulin give 50 ml dextrose 20%	→	Check after 20 minutes

Table S1. Results of univariate and multivariate analysis with glycemic lability index

	Univariate		Multivariate	
**Variable**	**Percentage of change in glycemic variability [95 % CI]**	**P–value**	**Percentage of change in glycemic variability [95 % CI]**	**P–value**
**APACHE II score < 15**				
Bolus infusion of hydrocortisone	25 [-13 – 79]	0.219	37 [-3 – 94][a]	0.076
**APACHE II score 15–24**				
Bolus infusion of hydrocortisone	28 [11 – 48]	< 0.001	36 [18 –57][b]	< 0.001
**APACHE II score > 24**				
Bolus infusion of hydrocortisone	19 [3 – 38]	0.022	13 [-6 – 36][c]	0.208

Abbreviations: APACHE, Acute Physiology and Chronic Health Evaluation; BMI, body–mass index; CI; confidence interval

[a]multivariate model includes the significant confounders Admission Type, and BMI

[b]multivariate model includes the significant confounders Admission Type, gender and BMI

[c]multivariate model includes the significant confounders Admission Type, and gender

Table S2. Results of multivariate analysis with insulin infusion rate variability

	Multivariate including glycemic variability (GLI)
**Variable**	
**APACHE II score < 15**	
Bolus infusion of hydrocortisone	4.3 [2.7 – 6.9][a]
**APACHE II score 15–24**	
Bolus infusion of hydrocortisone	4.0 [3.3 – 4.9]
**APACHE II score > 24**	
Bolus infusion of hydrocortisone	3.1 [2.6 – 3.7]

Abbreviations: APACHE, Acute Physiology and Chronic Health Evaluation; BMI, body–mass index; CI, confidence interval; GLI, glycemic lability index

[a]multivariate model includes the significant confounder BMI

Table S3. Results of multivariate analysis with glycemic variability including diabetic status as confounder

Variable	Multivariate model glycemic variability (SD)		Multivariate model glycemic variability (GLI)	
	**Percentage of change in glycemic variability [95 % CI]**	**P–value**	**Percentage of change in glycemic variability [95 % CI]**	**P–value**
**APACHE II score < 15**				
Bolus infusion of hydrocortisone	26 [8 – 47][a]	0.0034	43 [-6 – 116][a]	0.091
**APACHE II score 15–24**				
Bolus infusion of hydrocortisone	25 [18 – 34][b]	< 0.001	42 [20 – 69][b]	< 0.001
**APACHE II score > 24**				
Bolus infusion of hydrocortisone	11 [4 – 19][c]	< 0.003	12 [-6 – 35][c]	0.212

Abbreviations: APACHE, Acute Physiology and Chronic Health Evaluation; BMI, body–mass index; CI; confidence interval; GLI, glycemic lability index; SD, standard deviation

[a]multivariate model includes the significant confounders Admission Type, BMI and diabetic status

[b]multivariate model includes the significant confounders Admission Type, gender, BMI and diabetic status

[c]multivariate model includes the significant confounders Admission Type, gender and diabetic status

Table S4. Results multivariate analysis with insulin infusion rate variability including diabetic status

	Multivariate including diabetes	Multivariate including glycemic variability (SD) and diabetes	Multivariate including glycemic variability (GLI) and diabetes
**Variable**	**Odds Ratio [95%–CI]**	**Odds Ratio [95%–CI]**	**Odds Ratio [95%–CI]**
**APACHE II score < 15**			
Bolus infusion of hydrocortisone	3.6 [2.0 – 6.5]	3.7 [1.9 – 6.8][a]	4.2 [2.3 – 7.6][a]
**APACHE II score 15–24**			
Bolus infusion of hydrocortisone	3.8 [3.0 – 4.9]	3.3 [2.6 – 4.3]	3.8 [3.0 – 4.9]
**APACHE II score > 24**			
Bolus infusion of hydrocortisone	2.9 [2.3 – 3.7]	2.9 [2.2 – 3.6]	2.9 [2.3 – 3.7]

Abbreviations: APACHE, Acute Physiology and Chronic Health Evaluation; BMI, body–mass index; CI, confidence interval; GLI, glycemic lability index; SD, standard deviation

[a]multivariate model includes the significant confounder BMI
